# Transactions of the Buffalo Obstetrical Society

**Published:** 1885-07

**Authors:** 


### Transactions of the Buffalo Obstetrical Society.

*Stated Meeting, May 26, 1885.*

The President, Dr. W. W. Potter, in the chair; Dr. William H. Thornton, Secretary.

Dr. Charles G. Stockton read a paper on "The Treatment of Intestinal Diseases of Infancy." [See Original Communications, page 541.]

Dr. Thomas Lothrop most thoroughly and cordially endorsed the views expressed in the paper. He argued with the writer in the place of treatment proposed, based as it was upon strictly physiological principles, and stated that it was the greatest advance made since he had been in practice in the treatment of infantile diseases of the intestinal tract. He believed that the successful treatment of these cases, speaking generally, was more a question of management in regard to feeding than of mere drug-giving. When the profession would take this view and address itself to the perfection of infant feeding, he thought more good would be accomplished and better results obtained in reducing infant mortality.

Dr. C. C. Frederick had become familiar with the writer's views and with his plan of management in these cases, in the last year or two, from association with him in the case of a number of patients. He had put into practical use the treatment recommended, and found it, with scarcely an instance of failure, to prove efficient, and promptly so, even when other plans have been tried and failed. The plan of preparing the food according to his instructions might appear, at first, a little difficult, but with some practice and patient attention to details, most mothers and nurses soon acquired proficiency in this regard, and became quite expert in the preparation of the food.

Dr. F. H. Potter said that often a correct plan of action had been formulated, as had been so admirably done by the author of the paper, it only remained to obtain familiarity with its details to ensure success. Upon thoroughness in the technique, rested the successful application of the measure to practice. He thought it would be difficult or impossible to obtain the pure milk, insisted upon so properly by the writer as essential to the proper foundation of his line of treatment, from cows stabled and fed in the "cow barns" in this city. These distillery-fed animals certainly did not appear healthy, and he looked upon their milk with suspicion. He thought it very important for the doctor to examine into the sources of the milk supply when treating these cases with a view to the correction of errors at the fountain head.

Dr. P. W. Van Peyma was reminded by the paper of the fact, which he believed was admitted, that the children of the other countries, especially European, did not suffer with these diseases as much as children in America; that summer complaint, so-called, was more prevalent in the United States than in Europe. He did not pretend to offer any explanation of the difference, as he had never been able to determine the reasons for it, but simply spoke of it as interesting, and as having some bearing in explanation of our apparent want of success in the treatment of the diseases in question. He called attention to the influence which the nervous system exerted over these cases, and suggested the importance of carefully ministering to the nervous phenomena when they preponderated; also of guarding against sudden changes of temperature, and all things that predisposed to fermentation.

Dr. M. Hartwig, while he thoroughly enjoyed the philosophical and agreeably written paper, regretted that the writer had confined himself to the chronic forms of ill-nutrition, passing with mere mention that form of indigestion pertaining to breast-fed children. In regard to the means employed, he could see no good foundation for ipecac wine. He had observed certain advantages in the use of salicylic acid in very minute doses, to prevent fermentative changes. He thought there would be difficulty in carrying out the details of the writer's plan in poor families.

Dr. J. W. Keene had tried the plan of peptonizing milk, and had come to look upon it as a good deal in the line of the last new thing, which was the universal panacea. He had found difficulty in getting children to take peptonized milk on account of its taste ; thought it could only be properly prepared by an experienced person. He had employed condensed milk considerably, and found it to work satisfactorily, when properly prepared, in most instances ; though, of course, there were cases when that, or anything else for that matter, would not be well borne.

Dr. W. S. Tremaine was impressed with the unanimity of sentiment in the meeting, and how well the members agreed with Dr. Stockton, showing that his treatment must be correct. He was, however, surprised that the doctor had not given us the name, species, order and genus of the microbe that causes the diseases referred to ! This seemed to be so much the fashion now-a-days that its omission was alike notable and refreshing.

He corroborated Dr. Van Peyma's statement regarding the greater prevalence of infantile diarrhœa in this country than in Europe ; thought it might possibly be due to the preponderating influence of the nervous system over the physical organism. He thought the influence of long-continued hot weather, producing innervation, so that indigestion does not properly go on, might be one potent cause of these diseases. He believed in the use of calomel and gray powder in the treatment of infantile diarrhœas, to be given in the early stages. He had tried peptonized milk with adults, but found that, owing to its unpleasant taste, they would seldom take it long enough to be beneficial.

Dr. H. D. Ingraham agreed with the writer of the paper in regard to the use of peptonized milk. He had used it himself and was very much in favor of it, though he found it difficult oftentimes to get it properly prepared. His experience with the formula given for pancreation and soda was that the quantity of soda was too large. Did not have difficulty in getting children to take properly peptonized milk.

The President alluded to the fact that the mother's milk was frequently difficult in its watery constituents, and, as a consequence, contained an excess of solid matter. This was especially the case with the working mothers, who perspired excessively, and this was one reason why breast-fed children did not thrive. In each case, he would supply them with liberal quantities of pure water. He believed in the proposition of Dr. Van Peyma, reiterated by Dr. Tremaine, that intestinal diseases of infants are, if not caused, at least aggravated by some peculiar influence of the nervous system, rendering it difficult to treat them efficiently and successfully, unless this important factor was recognized and carefully ministered unto. He raised the question whether it was so much heat as sudden changes from cold to heat and *vice versa* that caused infantile diarrhœas. The mortuary records of the cities always exhibit a large number of deaths from cholera infantum, while, as a matter of fact, this was not a very common disease. He took the occasion to say a word against the careful manner in which death certificates were made up. There was a looseness of nomenclature which rendered health board statistics nearly valueless for purposes of scientific research. He referred to a paper by Dr. Alex. Hutchins on salicylate of calcium in serious diarrhœas, and expressed a favorable opinion in regard to the salicylates in these diseases. He opposed the use of the bromides as routine practice; they irritated the intestinal tract very frequently, besides increasing cerebral anæmia, a condition so desirable to overcome. He expressed very little faith in lime waters, so far as the alkaline properties were concerned, but was decidedly in favor of the bismuth treatment. He gave the latter in larger doses than usual—fed it in form of cream with water and gum arabic—the only limit being the amount a child will take when the case is extreme. He opposed the use of astringents as a rule, but favored the employment of anodynes to allay nervous perturbation, and cordially approved of the methods set forth in the paper as to artificial feeding. With pure milk, artificially digested when necessary, fresh air, wholesome water and scrupulous cleanliness, these cases were placed in as favorable a condition for recovery as possible, requiring very little medicine and much management.

Dr. Stockton, in closing the discussion, stated that he had employed peptonized milk in more than one hundred cases, having used it in every case when indicated, for two summers, and it was from this experience that his views had been given to the Society. He thought there was no difficulty in getting pure milk in Buffalo, and mentioned the supply from a number of cows as being preferable to the milk from one cow, the popular idea to the contrary notwithstanding. The long-continued use of artificial food of any kind, excepting milk, was certainly detrimental. He thought that failure in peptonizing milk was due to lack of careful or scientific attention. It was a matter that needed a little knack, and that was all there was of it. It should not be made too bitter, and he seldom found trouble in getting infants to take it, unless it became necessary to very thoroughly peptonize it, then they sometimes declined it on account of its bitterness. Believed in the importance of fresh air, and endorsed the bismuth treatment; but thought the nervous element probably the most important part of the whole subject.

